# Corneal lesions in erythema multiforme minor – Are systemic steroids indicated?

**DOI:** 10.4103/0301-4738.71701

**Published:** 2010

**Authors:** Shashi Jain, M K Rathore, P C Dwivedi, Eva Tirkey

**Affiliations:** Department of Ophthalmology, SS Medical College, Rewa – 486 001, Madhya Pradesh, India

Dear Editor,

We have read with interest the brief communication “Coin-shaped epithelial lesions following an acute attack of erythema multiforme minor with confocal microscopy findings” by Babu *et al*.[1] We appreciate that the authors have drawn attention to this relatively uncommon finding. Regarding the treatment of erythema multiforme (EM) minor, we would like to present few points.

EM is an acute and a self-limiting mucocutaneous hypersensitivity reaction associated with certain infections, medications, and other various triggers. EM minor is considered the mildest form of EM. It is characterized by skin eruption with or without mucosal involvement and may present with a wide spectrum of severity. Ocular involvement in EM minor is usually mild and may manifest as red conjunctivae, chemosis and lacrimation. We also recently came across a case of simultaneous presentation of bilateral coin-shaped discrete and few coalesced epithelial lesions with EM minor.

**Figure 1:**
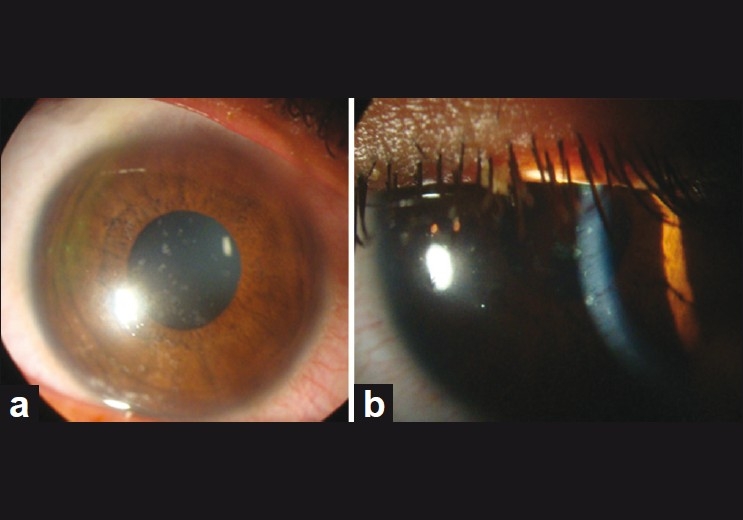
(a) Slit-lamp photograph of the cornea in diffuse illumination showing coin-shaped discrete and coalesced lesions with central clearing in some lesions. (b) Slit-lamp photograph in slit illumination showing discrete and coalesced coin-shaped epithelial lesions

**Figure 2:**
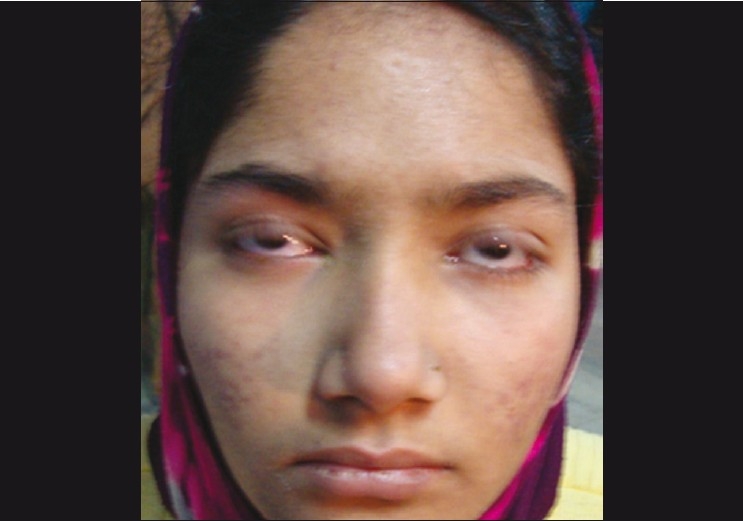
Photograph of the face showing papular skin lesions

**Figure 3:**
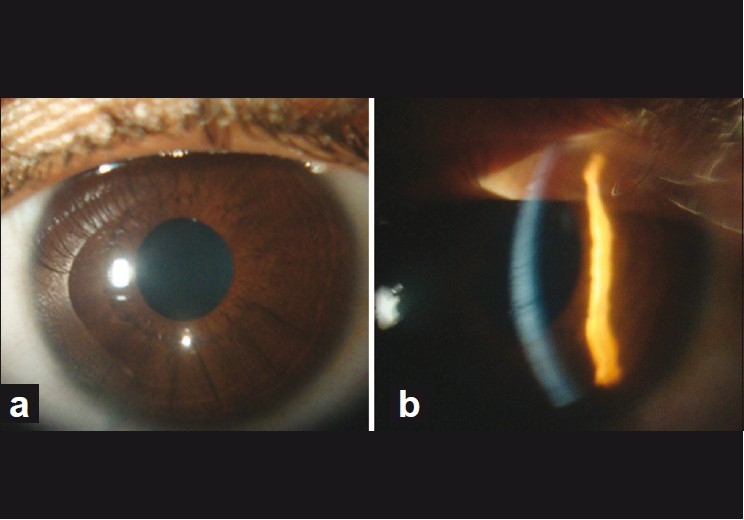
(a) Slit-lamp photograph showing disappearance of corneal lesions (diffuse illumination). (b) Slit illumination showing disappearance of corneal lesions

A 17-year- old female presented with sudden diminution of vision in both eyes since 5 hrs. She had watering and irritation in both eyes along with a history of cold and cough for 3 days. There was no history of any drug intake. On ophthalmic examination, her best-corrected visual acuity was 20/40 in both eyes. Slit-lamp examination in both eyes revealed multiple coin-shaped epithelial lesions, few coalesced and some showing central clearing [Fig. 1a and b]. There was no involvement of stroma. Corneal sensation and the rest of anterior segment and posterior segment were normal. On general inspection, she had multiple papular rashes on face, arms, and legs [Fig. 2] for which she was advised to consult a dermatologist, who diagnosed EM minor, and advised symptomatic treatment. The erythrocyte sedimentation rate was 45 mm/h and total white blood count was 11,000 cells/mm^3^ with predominant neutrophils (70%). For corneal lesions, she was prescribed topical steroid drops four times a day along with lubricant drops and acyclovir eye ointment 3% three times a day. Corneal lesions resolved within a week. Visual acuity improved to 20/20 and slit-lamp examination showed normal corneal epithelium [Fig. 3a and b].

In the literature (Medline search),[2–4] it is described that for all forms of EM, no specific treatment is available but the most important treatment is usually symptomatic supportive care, with the identification and removal of the trigger factor. Systemic corticosteroid therapy is controversial and has no effect on the severity of ocular manifestations and prognosis. Some believe it may predispose to complications. EM minor is typically asymptomatic and the lesions may clear up themselves within 2–3 weeks even without treatment.

We report this case to highlight that epithelial lesions associated with EM minor have responded well to topical steroid drops and completely resolved in a week’s time.
